# Supplementary material for: Design of a Lab-On-Chip for Cancer Cell Detection through Impedance and Photoelectrochemical Response Analysis
Source: Biosensors (Basel). 2022 Jun 13;12(6):405. doi: 10.3390/bios12060405 (PMC9221223; doi:10.3390/bios12060405)
Supplement: Supplementary file 1 [file biosensors-12-00405-s001.zip › biosensors-1732731-supplementary.pdf]

biosensors

MDPI

Article

Design of a Lab-On-Chip for Cancer Cells Detection Through Impedance and Photoelectrochemical Response Analysis: Supplement Document

Yu-Ping Hsiao [1,2], Arvind Mukundan [3], Wei-Chung Chen [4], Ming-Tsang Wu [4,5,6,7], Shang-Chin Hsieh [8,*], and Hsiang-Chen Wang [3,*]

[1] Department of Dermatology, Chung Shan Medical University Hospital, No.110, Sec. 1, Jianguo N. Rd., South District, Taichung City 40201, Taiwan; cshy713@csh.org.tw
[2] Institute of Medicine, School of Medicine, Chung Shan Medical University, No.110, Sec. 1, Jianguo N. Rd., South District, Taichung City 40201, Taiwan
[3] Department of Mechanical Engineering, Advanced Institute of Manufacturing with High Tech Innovations (AIM-HI), Center for Innovative Research on Aging Society (CIRAS), National Chung Cheng University, 168, University Rd., Min Hsiung, Chia Yi 62102, Taiwan; d09420003@ccu.edu.tw
[4] Ph.D. Program in Environmental and Occupational Medicine, Kaohsiung Medical University, Kaohsiung 80708, Taiwan; u103803001@kmu.edu.tw (W.-C.C.); mingtswu@gmail.com(M.-T.W.)
[5] Research Center for Environmental Medicine, Kaohsiung Medical University, Kaohsiung 80708, Taiwan
[6] Department of Public Health, Kaohsiung Medical University, Kaohsiung 80708, Taiwan
[7] Department of Family Medicine, Kaohsiung Medical University Hospital, Kaohsiung Medical University, Kaohsiung 80708, Taiwan
[8] Department of Plastic Surgery, Kaohsiung Armed Forces General Hospital, 2, Zhongzheng 1 st. Rd., Lingya District, Kaohsiung City 80284, Taiwan
* Correspondence: sschin522@yahoo.com.tw (S.-C.H.); hcwang@ccu.edu.tw (H.-C.W.)

Citation: Hsiao, Y.-P.; Mukundan, A.; Chen, W.-C.; Wu, M.-T.; Hsieh, S.-C.; Wang, H.-C. Design of a Lab-On-Chip for Cancer Cells Detection Through Impedance and Photoelectrochemical Response Analysis: Supplement Document. *Biosensors* **2022**, *12*, 405. https://doi.org/10.3390/bios12060405

Received: 3 May 2022
Accepted: 9 June 2022
Published: 13 June 2022

Publisher's Note: MDPI stays neutral with regard to jurisdictional claims in published maps and institutional affiliations.

Copyright: © 2022 by the authors. Submitted for possible open access publication under the terms and conditions of the Creative Commons Attribution (CC BY) license (https://creativecommons.org/licenses/by/4.0/).

Abstract: This document provides the Supplementary Materials to the article Design of a Lab-On-Chip for Cancer Cells Detection Through Impedance and Photoelectrochemical Response Analysis. Section 1 briefly describes about the apparatus used in this study. Section 2 illustrates about the design of the microelectrodes. Section 3 gives a brief overview of flowchart of the chip fabrication process. Section 4 provides the results of the photoelectric response measurement. Section 5 gives the calculation formula for cell admittance measurement while Section 6 gives a brief overview of how the cells were counted. Finally the last section gives the tables of regression analysis of different cell types.

Keywords: Lab-on-chip; linear interdigitated sawtooth electrode; dielectrophoretic impedance, photocurrent response measurement; electron beam evaporation; electrode lithography process

1. Apparatus Description

The Microtest 6630 Precision Impedance Analyzer was used in this study along with a Function generator (Agilent 33220 A) to measure with a 1 Vp-p voltage AC frequency, because the signal generator can provide excitation signals with a wide range of amplitudes and frequencies. An upright microscope (OLYMPUS, BX43) is connected with a CCD lens (DP71, Olympus, Tokyo, Japan) to a computer to observe and record changes in cell samples Schematic. A micro-galvanometer (AMETRIX Instruments Model 101) was used as the primary measurement of a photoelectric flow measurement system provides resolution and accuracy superior to traditional more expensive instruments and provides quiet, stable measurements with a substantial reduction in measurement time. The photoelectric flow measurement system fixes the needle pressure and lower needle position of the probe through a micropositioning probe seat (Microposi-

tioner EB-700). The software (Pluse Scan V1.1) is provided by the manufacturer and written by LabVIEW RTE2013. Figure S1 shows schematic diagram of impedance measurement system and Figure S2 shows the schematic diagram of photoelectric flow measurement system.

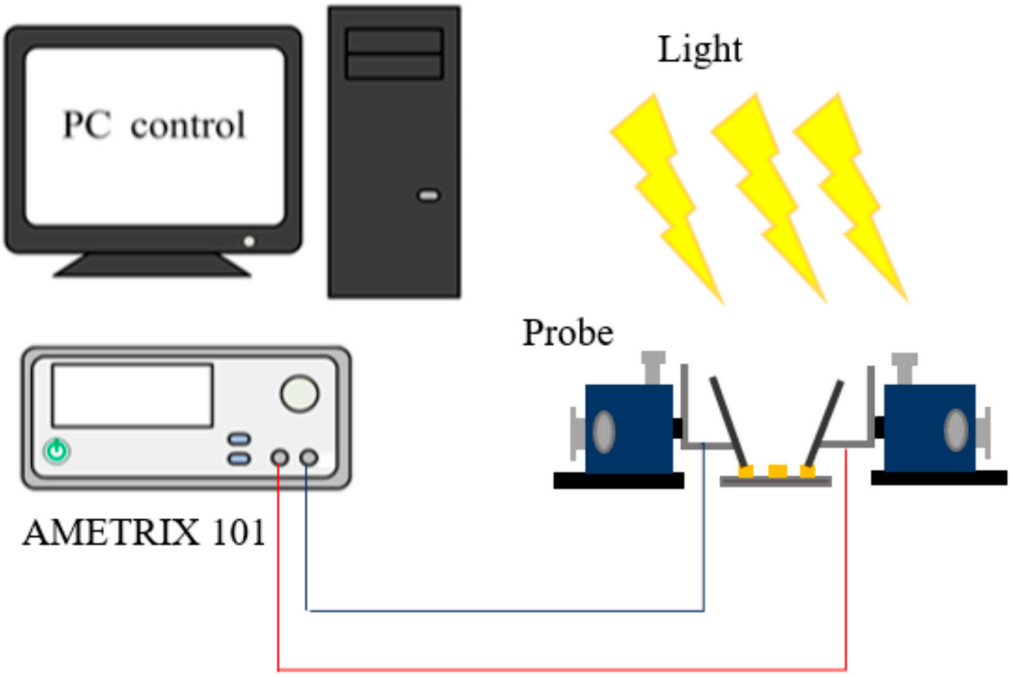

Figure S1. Schematic diagram of impedance measurement system.

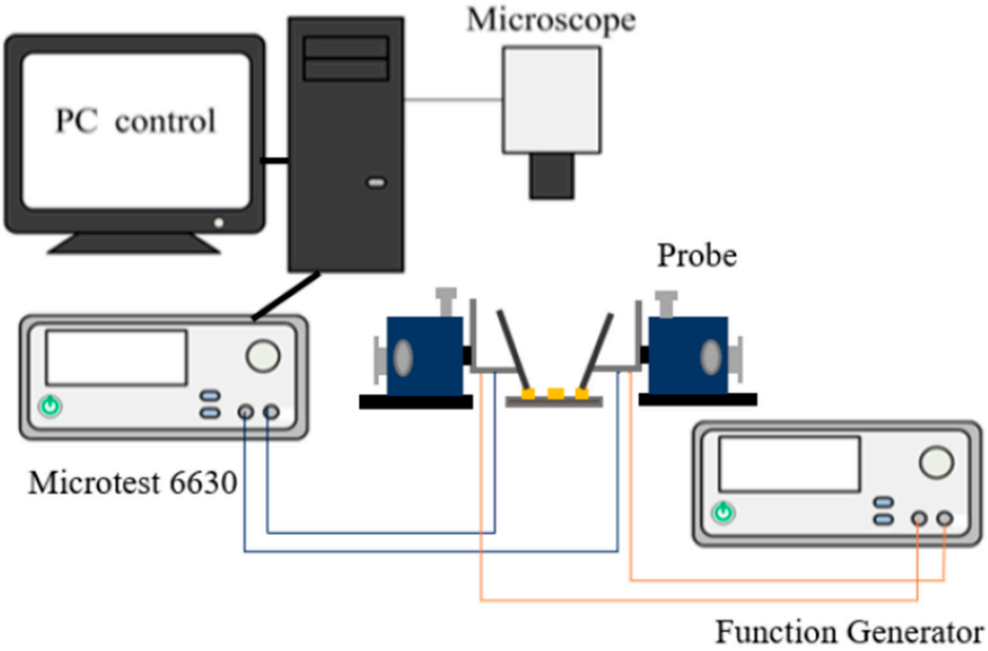

Figure S2. Schematic diagram of impedance measurement system.

2. Design of the microelectrodes

Figure S3 shows the schematic diagram of the biosensor chip architecture while Figure S4 shows the schematic diagram of microelectrodes under OM.

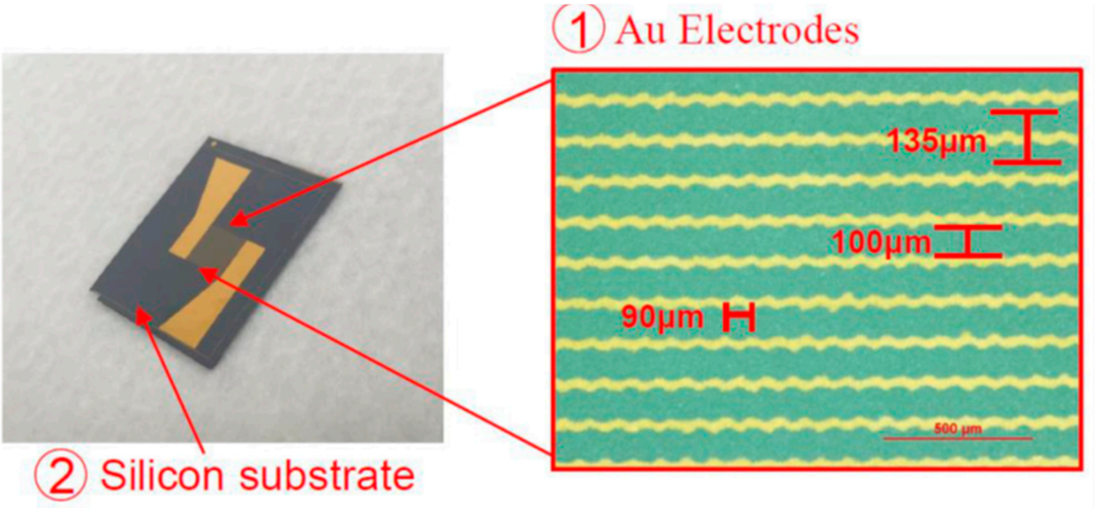

Figure S3. Schematic diagram of biosensor chip architecture.

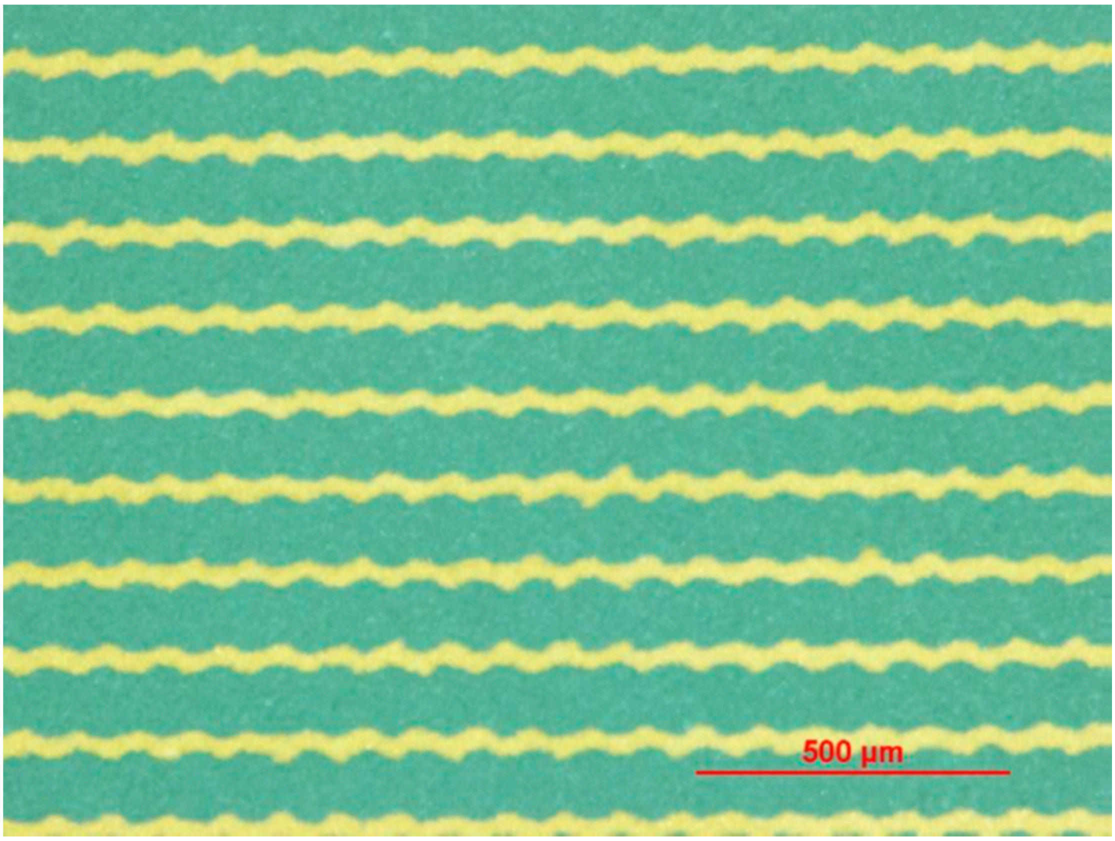

Figure S4. Schematic diagram of microelectrodes under OM.

3. Chip fabrication

Figure S5 shows the flow chart of biosensor chip fabrication while Figure S6 is the actual finished product of the biosensor chip.

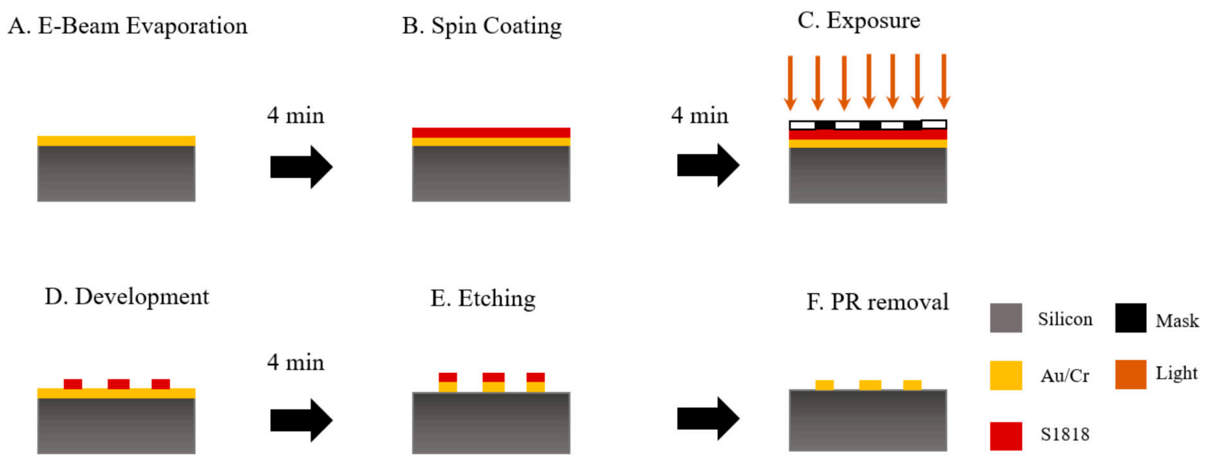

Figure S5. The flow chart of biosensor chip fabrication.

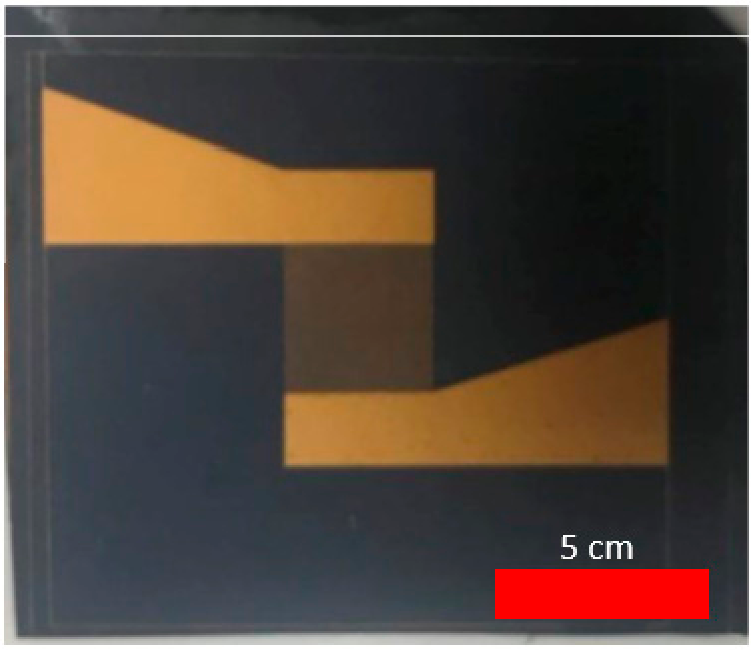

Figure S6. Actual finished product of the biosensor chip.

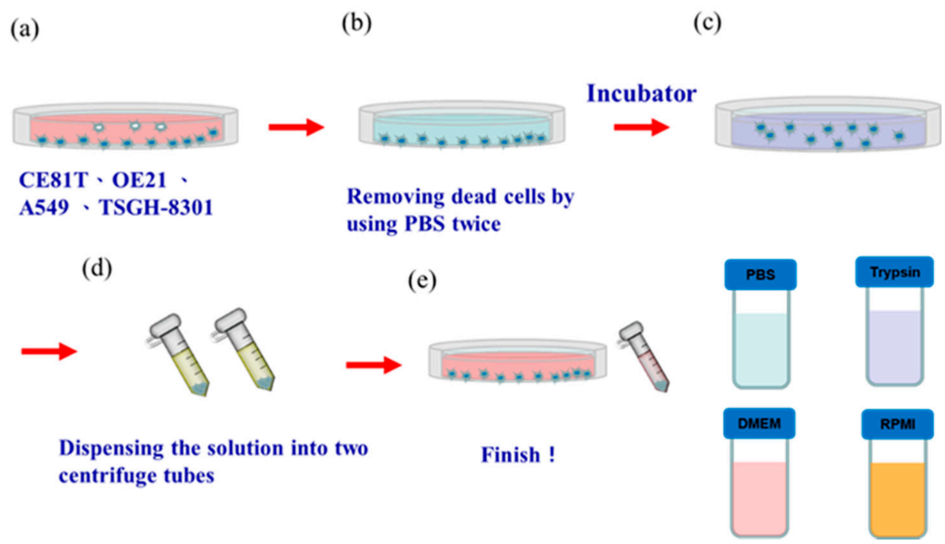

Figure S7. Schematic diagram of reparation of the different cells.

4. Photoelectric Response Measurement

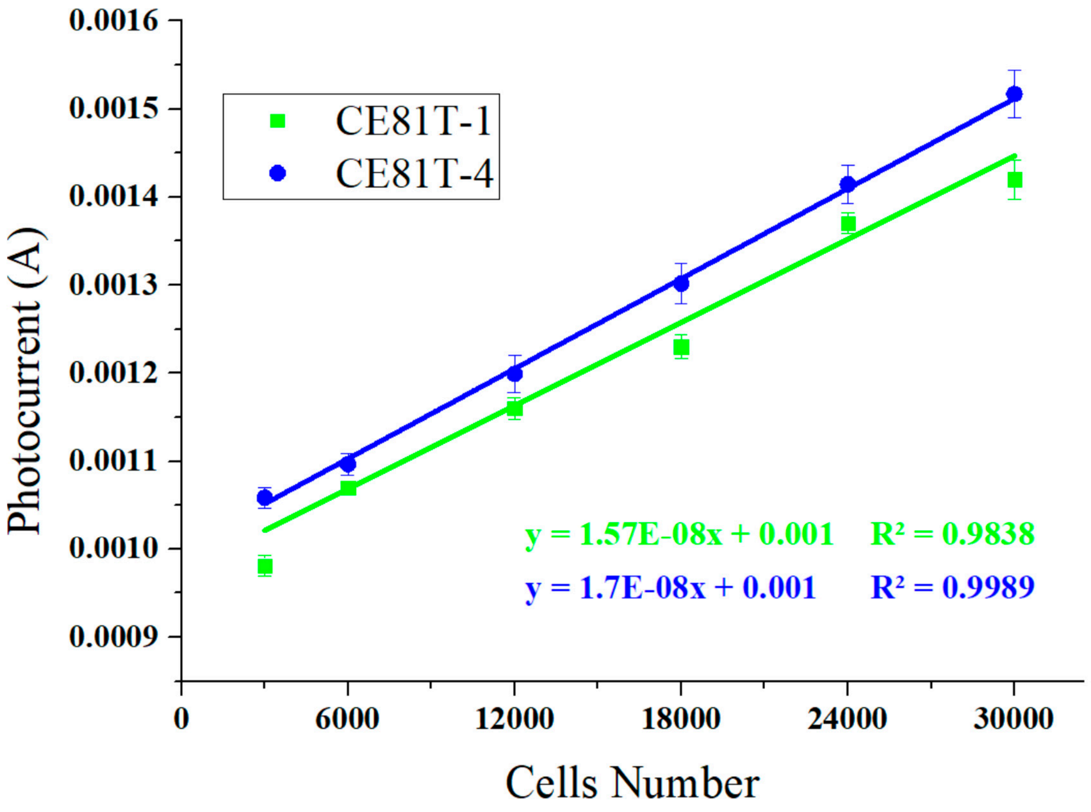

Figure S8. The photoelectric flow measurement result of CE81 T esophageal cancer cells under different cell numbers, among which CE81 T-1 is the first stage cancer lesion, CE81 T-4 is the fourth stage cancer lesion.

5. Calculation formula for cell admittance measurement

After the cells are aggregated by applying positive dielectrophoresis on the microelectrode, physical parameters such as impedance, admittance, capacitance, and conductance value will change after the cells are grasped by the electrode. In this study, the impedance value was measured by an impedance analyzer. The transformation of the electrical signal during the measurement will be converted into an exact impedance value,

and when the cells are aggregated by the dielectrophoretic force, the cells are connected in parallel between the two electrodes in a string of pearls, so a parallel module is used in the cell impedance measurement. The total impedance Z_A of the electrode is divided into two parts, the real part and the imaginary part, as shown in Equation (S1). Among them, Z_A is the total impedance (cell impedance + electrode impedance), R_A is the total resistance (cell resistance + electrode resistance), C_A is the total capacitance (cell capacitance + electrode capacitance). When $R_0>>Z_A$, Equation (S2) is used with V_0 as the initial voltage measurement.

$$Z_A = \frac{R_A}{1+\omega^2 {C_A}^2 {R_A}^2} + \frac{-\omega C_A {R_A}^2}{1+\omega^2 {C_A}^2 {R_A}^2} j \quad \text{(S1)}$$

$$u_1 = \frac{Z_A}{R_0} \times V_0 e^{j\omega t} \quad \text{(S2)}$$

The voltage value measured by the lock-in amplifier $u_2 = u_0 e^{(\omega t+\theta)}$, where u_0 is the partial pressure of the lock-in amplifier received through the electrode, and $\omega t + \theta$ is the phase angle generated by the voltage passing through the electrode and the cell. The experiment is mainly based on the most idealized results, so assuming $u_1 = u_2$ and using Equation (S3),

$$Z_A = \frac{R_0}{V_0} u_0 \cos\varphi + j \frac{R_0 u_0 \sin\varphi}{v_0} \quad \text{(S3)}$$

Substitute the Equation (S1) into Equation (S3) for equation operation, and then comparing and solving the real and imaginary parts of the formula separately, we get the Equation (S4) and Equation (S5).

$$R_A = \frac{R_0 u_0 \sin\varphi}{V_0} \times \frac{(1+\tan^2\varphi)}{\tan\varphi} \quad \text{(S4)}$$

$$C_A = \frac{\text{-}V_0}{\omega R_0 u_0 \sin\varphi} \times \frac{\tan^2\varphi}{1+\tan^2\varphi} \quad \text{(S5)}$$

In this study, R_E is the electrode resistance, R_T is the pearl string resistance, C_E is the electrode capacitance, C_T is the cell pearl string capacitance, S_E is the electrode conductance, S_T is the cell pearl string admittance. The cell and the electrode are connected in parallel, so according to Ohm's law the sum of the resistances, capacitance is given in Equation (S6) and Equation (S7) respectively.

$$R_T = \frac{R_E R_A}{R_E - R_A} \quad \text{(S6)}$$

$$C_T = C_A - C_E \quad \text{(S7)}$$

The signal measured in this experiment is finally empirically calculated to obtain as given in Equation (S8).

$$S_T = S_A - S_E \quad \text{(S8)}$$

6. Cell count

As shown in Figure S9, there are two nine-square grids on the counting plate, and each nine-square grid is engraved with 9 small squares of 1 mm^2. The small squares located at the 4 corners are further carved into 16 small grids, each with a height of 0.1

mm. The volume between each slide and the counting plate is 1 mm^2 × 0.1 mm = 1.0 × 10$^{–4}$ ml. Before used it is disinfected with 95% alcohol. 10 μl of cell fluid and 10 μl of trypan blue are taken and mixed evenly. 10 μl from each pipette is taken and add it from the groove and cover it with a glass and observe it under a microscope. As shown in Figure S1 when counting, divide the number of cells in the upper and lower eight squares by 8, multiply by the dilution factor and finally multiply by 104, which is the number of cells suspended per milliliter of cells. If the number of cells is too large, it can be diluted with sucrose solution and then injected to re-count; otherwise, centrifuge (500 rpm/5 min) and remove the supernatant for aggregation and re-count.

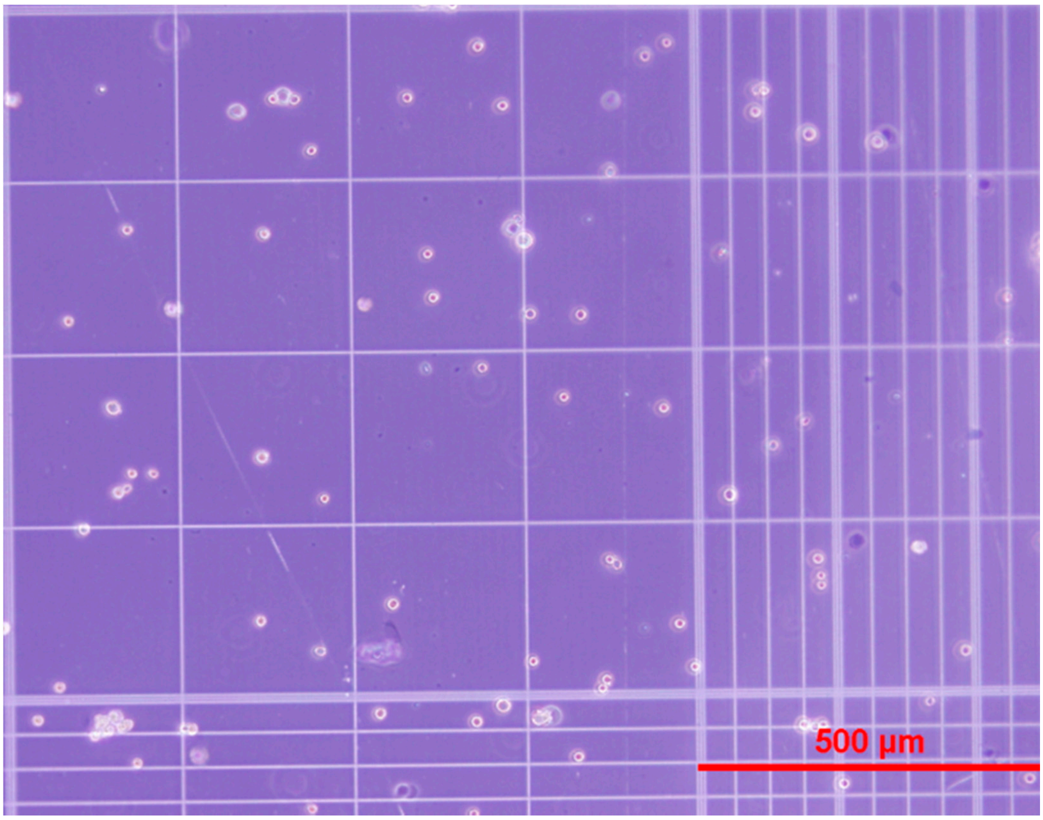

Figure S9. Under the microscope, live cells appear white, and dead cells appear blue.

7. Regression Analysis

LOD was calculated based on the standard deviation of the Slope. It can be expressed as three times of the SD of the slope divided by the slope. While limit of quantification can be calculated by times of the SD of the slope divided by the slope and the sensitivity is calculated by dividing the slope of linearity graph by the geometry or active area of biosensor.

Table S1. Regression Analysis of A549.

A549

Regression Statistics	
Multiple R	0.993544994
R Square	0.987131655
Adjusted R Square	0.983914569
Standard Error	0.000192219
Observations	6

ANOVA

	df	*SS*	*MS*	*F*	*Significance F*
Regression	1	1.13372E-05	1.13372E-05	306.8402794	6.23662E-05
Residual	4	1.47793E-07	3.69482E-08		
Total	5	1.1485E-05			

	Coefficients	*Standard Error*	*t Stat*	*P-value*	*Lower 95%*	*Upper 95%*	*Lower 95.0%*	*Upper 95.0%*
Intercept	0.000614682	0.000149571	4.109642245	0.014736453	0.000199407	0.001029956	0.000199407	0.001029956
X Variable 1	1.439E-07	8.21494E-09	17.51685701	6.23662E-05	1.21092E-07	1.66708E-07	1.21092E-07	1.66708E-07

Table S2. Regression Analysis of TSGH-8301.

TSGH-8301

Regression Statistics	
Multiple R	0.99755475
R Square	0.99511548
Adjusted R Square	0.993894349
Standard Error	8.22085E-05
Observations	6

ANOVA

	df	*SS*	*MS*	*F*	*Significance F*
Regression	1	5.50738E-06	5.50738E-06	814.913545	8.96156E-06
Residual	4	2.7033E-08	6.75824E-09		
Total	5	5.53441E-06			

	Coefficients	*Standard Error*	*t Stat*	*P-value*	*Lower 95%*	*Upper 95%*	*Lower 95.0%*	*Upper 95.0%*
Intercept	0.000362468	6.39685E-05	5.666343638	0.004783499	0.00018486	0.000540073	0.000184863	0.000540073
X Variable 1	1.00295E-07	3.51338E-09	28.54669062	8.96156E-06	9.0541E-08	1.1005E-07	9.05406E-08	1.1005E-07

Table S3. Regression Analysis of CE81 T-1/VGH.

CE81T-1/VGH

Regression Statistics	
Multiple R	0.998573281
R Square	0.997148598
Adjusted R Square	0.996435748
Standard Error	8.54144E-05
Observations	6

ANOVA

	df	*SS*	*MS*	*F*	*Significance F*
Regression	1	1.02052E-05	1.02052E-05	1398.818772	3.05184E-06
Residual	4	2.91825E-08	7.29562E-09		
Total	5	1.02344E-05			

	Coefficients	*Standard Error*	*t Stat*	*P-value*	*Lower 95%*	*Upper 95%*	*Lower 95.0%*	*Upper 95.0%*
Intercept	0.000670376	6.64631E-05	10.08644211	0.00054358	0.000485845	0.000854908	0.000485845	0.000854908
X Variable 1	1.36527E-07	3.65039E-09	37.40078572	3.05184E-06	1.26392E-07	1.46662E-07	1.26392E-07	1.46662E-07

Table S4. Regression Analysis of OE21–1/VGH.

OE21-1/VGH

Regression Statistics	
Multiple R	0.997461968
R Square	0.994930378
Adjusted R Square	0.993662972
Standard Error	0.000148424
Observations	6

ANOVA					
	df	*SS*	*MS*	*F*	*Significance F*
Regression	1	1.72936E-05	1.73E-05	785.0134	9.65423E-06
Residual	4	8.81189E-08	2.2E-08		
Total	5	1.73818E-05			

	Coefficients	*Standard Error*	*t Stat*	*P-value*	*Lower 95%*	*Upper 95%*	*Lower 95.0%*	*Upper 95.0%*
Intercept	0.000610247	0.000115493	5.283858	0.006154	0.000289588	0.000930906	0.000289588	0.000930906
X Variable 1	1.77726E-07	6.34326E-09	28.01809	9.65E-06	1.60114E-07	1.95338E-07	1.60114E-07	1.95338E-07

Author Contributions: Conceptualization, S.-C.H., Y.-P.H., W.-C.C., and H.-C.W.; methodology, A.M., W.-C.C., Y.-P.H., M.-T.W., and S.-C.H.; software, S.-C.H., H.-C.W., and A.M.; validation, S.-C.H., W.-E.E., Y.-P.H., M.-T.W., and H.-C.W.; formal analysis, S.-C.H., Y.-P.H., and H.-C.W.; investigation, S.-C.H. and M.-T.W.; resources, S.-C.H. and H.-C.W.; data curation, A.M., H.-C.W., Y.-P.H., and M.-T.W.; writing—original draft preparation, A.M.; writing—review and editing, A.M. and H.-C.W.; supervision, Y.-P.H. and H.-C.W.; project administration, H.-C.W. All authors have read and agreed to the published version of the manuscript.

Funding: This research was supported by the Ministry of Science and Technology, the Republic of China under grants MOST 105-2923-E-194-003 MY3, 108-2823-8-194-002, 109-2622-8-194-001-TE1, and 109-2622-E-194-007. This work was financially or partially supported by the Advanced Institute of Manufacturing with High-tech Innovations (AIM-HI) and the Center for Innovative Research on Aging Society (CIRAS) from the Featured Areas Research Center Program within the framework of the Higher Education Sprout Project by the Ministry of Education (MOE), Kaohsiung Medical University Research Center Grant (KMU-TC109A01), and Kaohsiung Armed Forces General Hospital research project 110-016 in Taiwan.

Institutional Review Board Statement: Institutional Review Board Statement: The study was conducted according to the guidelines of the Declaration of Helsinki, and approved by the Institutional Review Board of Kaohsiung Medical University Hospital (KMUH) (KMU-HIRB-G(II)-20200027).

Informed Consent Statement: Written informed consent was waived in this study be-cause of the retrospective, anonymized nature of study design.

Data Availability Statement: Data sharing not applicable.

Conflicts of Interest: The authors declare no conflict of interest.
